# Decision support at home (DS@HOME) – system architectures and requirements

**DOI:** 10.1186/1472-6947-12-43

**Published:** 2012-05-28

**Authors:** Michael Marschollek

**Affiliations:** 1 Hanover Medical School, Peter L. Reichertz Institute for Medical Informatics, Carl-Neuberg-Str. 1, Hanover , 30625, Germany

## Abstract

**Background:**

Demographic change with its consequences of an aging society and an increase in the demand for care in the home environment has triggered intensive research activities in sensor devices and smart home technologies. While many advanced technologies are already available, there is still a lack of decision support systems (DSS) for the interpretation of data generated in home environments. The aim of the research for this paper is to present the state-of-the-art in DSS for these data, to define characteristic properties of such systems, and to define the requirements for successful home care DSS implementations.

**Methods:**

A literature review was performed along with the analysis of cross-references. Characteristic properties are proposed and requirements are derived from the available body of literature.

**Results:**

79 papers were identified and analyzed, of which 20 describe implementations of decision components. Most authors mention server-based decision support components, but only few papers provide details about the system architecture or the knowledge base. A list of requirements derived from the analysis is presented. Among the primary drawbacks of current systems are the missing integration of DSS in current health information system architectures including interfaces, the missing agreement among developers with regard to the formalization and customization of medical knowledge and a lack of intelligent algorithms to interpret data from multiple sources including clinical application systems.

**Conclusions:**

Future research needs to address these issues in order to provide useful information – and not only large amounts of data – for both the patient and the caregiver. Furthermore, there is a need for outcome studies allowing for identifying successful implementation concepts.

## Background

Demographic change is induced and influenced by many factors. One of them is the desirable rising life expectancy, which is in turn due to e.g. better nutrition and improved medical care. The shift towards an elderly population with a significant proportion of people aged above 65 however also goes along with the challenge to maintain a stable level of care and care giving, knowing that the older a person becomes, the more likely she or he is to suffer from multiple chronic diseases. The Berlin Aging Study e.g. has shown that the prevalence rate of five or more somatic diseases was 88% in the group of persons aged 70 years and above [1]. At the same time, the primary – and well comprehensible – wish of elderly persons is to remain in their homes as long as possible despite a possible need for professional care.

Bearing this in mind, health-enabling technologies, and smart home technologies in particular, have been identified as potential measures to alleviate the consequences that demographic change will effect on societies. These technologies offer support in terms of ‘patient empowerment', i.e. in a person’s own health management [2], making more health-related data available than ever before. By handing over a certain amount of responsibility to the person, she or he is supposed to be put in a position to make informed decisions in health care matters. On the other hand, care givers and physicians may profit from smart home technologies by getting more long-term information about a patient, enabling him to make his decisions on a broader basis of information.

Despite several large-scale funding schemes and a multitude of very successful projects that have been conducted, trial-based evidence about the long-term effects of smart home technologies on the quality of care and the quality of life is still scarce [3-5]. One reason for this lack of evidence may be that the multitude of valuable data about a person’s state of health cannot be analyzed and aggregated in a way that relevant information for caregiver, physician or patient is extracted, and that technology is not fully integrated in care workflows [6].

In clinical settings, data analytics and decision support systems (DSS) have been established in several data-rich environments, most notably e.g. in ECG interpretation [7] or in mechanical ventilation in intensive care units [8].

Considering the rising relevance of home-based, health-enabling technologies, the overall aim of the research for this paper is to investigate and present the state-of-the-art in decision support systems for health care purposes or patient support in home environments, and in particular

· to define characterizing properties for these systems with regard to their dispersion, architectures and integration into home care (aim#1) and apply these to the results of a literature analysis, and

· to define the requirements for home care decision support systems with regard to architectural and functional aspects (aim#2).

The rest of this paper is arranged as follows: The “Methods” section describes the methods used in the literature search and analysis. Within the “Results” section, the literature review results are presented, followed by a sub-section proposing a categorization scheme for the systems identified. Subsequently, the identified systems are described in more detail with regard to their properties and specific features. The “Results” section concludes with a sub-section defining requirements for decision support systems in the context of home environments. The results are discussed critically and the paper concludes with a brief summary and a suggestion for future lines of research.

## Methods

The author has conducted a literature analysis in the PubMed/Medline database on June 17, 2011, using the search term

(‘decision support’ or ‘self-management’) *and*

(‘home’) *and*

(‘sensors’ or ‘smart’ or ‘tele’ or ‘telecare’ or ‘telemedicine’).

The abstracts of all research papers found during the search were reviewed with regard to their relevance for the aims of the study and using the following inclusion criteria:

· description of a dedicated decision support component,

· comprehensible implementation of a DS component, and

· application in a home environment.

**Figure 1:**
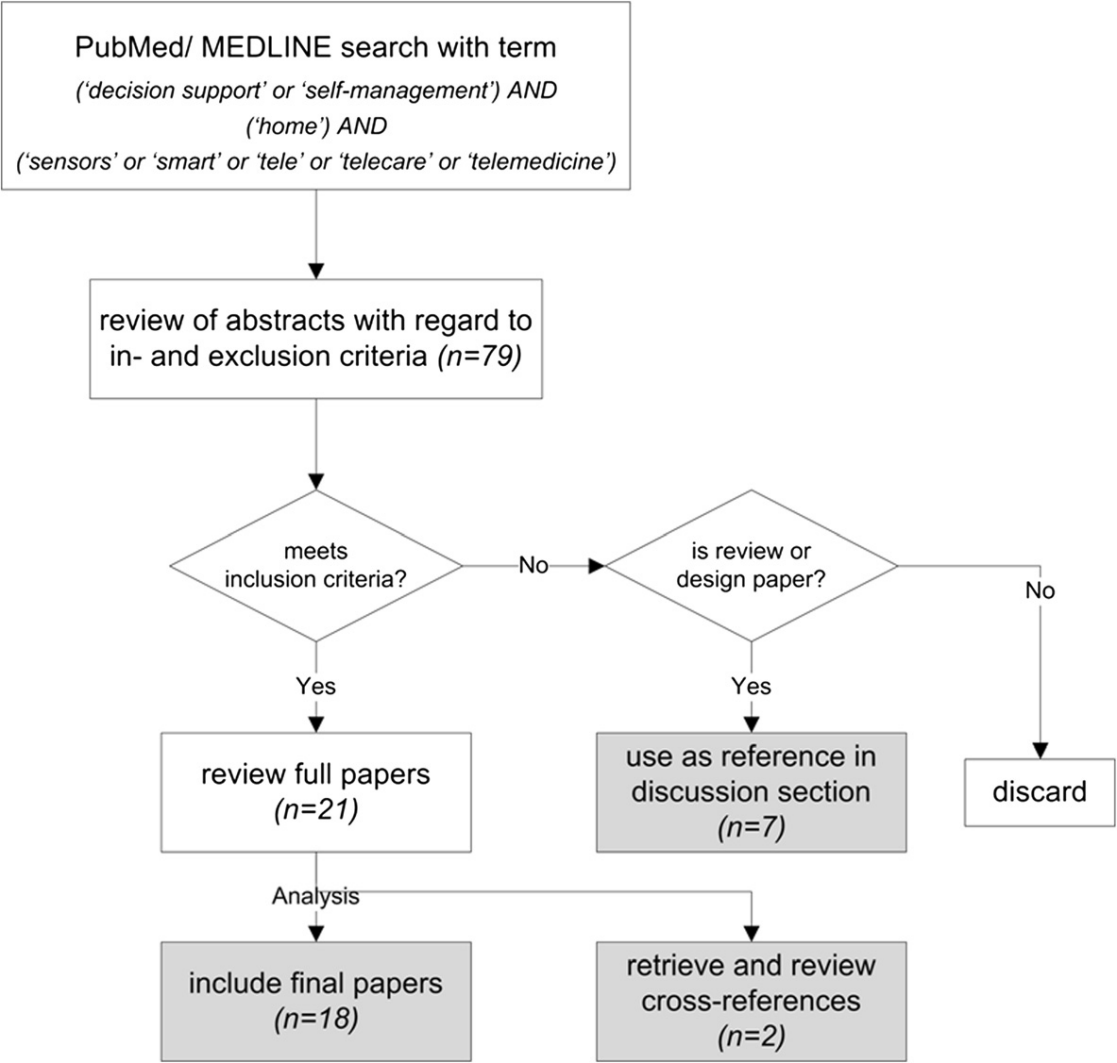
**Literature search and review method.** Altogether 27 papers have been retrieved in the process.

Subsequently, selected full papers meeting the inclusion criteria were analyzed (Figure 1). Apart from the guided search, cross-references within these papers were analyzed. Subsequently, all identified decision support systems in home environments were characterized using three basic properties proposed by the author (Table 1). Finally, using the available body of literature, the author has defined the requirements of such systems. Review or design papers identified during the literature search were included as references in the discussion section.

**Table 1:** Three main properties of the decision support systems identified

**Reference(s)**	**Location**		**Autonomy of decision component**				**HIS integration**			**remarks**
	**home**	**server**	**descript.analysis**	**single parameter**	**multi-parameter**	**fully autonomous, real-time**	**no**	**forwarding**	**full integration**	
Basilakis[9,10]		X			X			X	(X)	HIS integration plans mentioned
Bellazzi [11]		X		X				X		
Biddiss [12]		X?	X					X		telephone-based
Black [13]		X		X	?			X		
Bosworth [14]		X			X				X	(study design)
Cross [15,16]		X		X				X		cf. Joshi, Finkelstein
Finkelstein [17-20]		X		X	X			X		cf. Cross, Joshi
Helmer [21]	X				X			X		HIS integration plans mentioned
Joshi [22]		X		X				X		cf. Cross, Finkelstein
Marschollek [23,24]	X				X			X		HIS integration plans mentioned, cf. Song, Helmer
Reiter [25]	X	X			X			X		design description
Song [26]	X			X				X		HIS integration plans mentioned, cf. Helmer, Marschollek
Zheng [27,28]	X	X			X			X		

The references are sorted in alphabetical order by the first author’s names.

Complying with the rules of Good Scientific Practice [29], all search results may be requested from the author.

## Results

### Literature review

The literature search in the PubMed/Medline database yielded 79 hits, of which 18 met the inclusion criteria. Two further papers were identified by analyzing cross-references, and seven review papers were used for the results and discussion sections (Figure 1). The systems identified showed a wide variation in terms of autonomy, integration into existing health information system (HIS) structures, ability to provide patient feedback, number of parameters considered in decision making, and complexity (Table 1).

All of the articles identified as relevant describe an approach of data forwarding, sometimes after preprocessing/ preparation. Despite several published ideas concerning the integration of home decision support with health information system components (e.g. [14,23,25]) and a partial implementation [9,10], the author could not identify a fully integrated system.

13 out of 20 papers present multi-parametric data analysis components, six a single-parameter approach and one a descriptive analysis. No system performing autonomous decision-making in real-time was identified. Seven articles mention the deployment of at least a part of decision component in the actual home environment, yet the majority (16) places it on a central server unit (duplicate entries included).

### Categories of home care DSS

Based on the analysis of current DSSs which have been used or are in use in the context of care in home environments, the author proposes three main properties to denote the divergent mass of current systems.

1. *Location of the DSS component*: The analysis modules containing the medical logic, formally represented e.g. by rules [9,13,21,27] or a Bayesian network [30], may be located on a central server system to which the data are transferred [12,15] – maybe even in a clinical environment – or at the patient’s home [21]. The location has implications with regard to the possibility to update or change the modules [31], for example if new knowledge/ guidelines are released or if a doctor wants to adjust default modules in order to individualize treatment.

2. *Autonomy of the DSS*: The system’s tasks may range from descriptive analysis (for example graphical presentation of raw data) via various steps of data reduction and information extraction to support experts in their decision making to nearly autonomous real-time decision systems [30].

3. *Degree of HIS integration and adaptiveness*:The DSS may be isolated from other application systems, if e.g. to analyze blood glucose values or trends at home, but it may also be connected to clinical information systems or a GP practice [10], tapping EHR data to refine analyses and recommendations based on additional medical context knowledge.

These three properties were used to assess and categorize the current range of DSS in home care, but other categories might be equally helpful. Table 1 shows the results of this categorization.

### Relevance of DSS in home care

The importance of DSSs in the context of home care is stressed by five of the papers identified. Speedie et al. state that home-based physiological data can only be used to its full potential if clinical information from electronic health records (EHRs) is incorporated in the decision support process [32]. This point is also made by Lymberis [33] and Koch, who states that research in ‘better integration of new knowledge about treatment into evidence-based decision support tools at the point of care’ ([34], p. 572) is critical in the context of home-telehealth (see also [35]). Hermens and Vollenbroek-Hutten furthermore stress that – by employing methods of artificial intelligence – patient feedback can be provided, but that this area still lacks more intensive research [36].

Only very few authors have actually published (parts of) their knowledge base, mostly in the form of production rules. Examples of published knowledge bases can be found in [12,13,30,37]. The author has found even fewer reports aiming to standardize the decision logic, for example by using the Arden Syntax for Medical Logic Modules [21,37,38].

### Single- and multi-parameter analysis systems

Bellazzi et al. provide a good example of how advanced analysis methods (time series analysis/ decomposition, Bayesian methods, temporal abstraction) can be used to interpret blood glucose data series for decision support in diabetes patients [11].

Among the six articles presenting systems for decision-making based on single parameter data in the context of specific diseases, the ‘Home Automated Telemanagement system’ (HAT) described by Finkelstein et al. [17] provides a good example. It consists of one or more home units, a HAT server and a clinician unit. The patient data are collected from home-based devices for self-testing, for example pulmonary parameters relating to asthma management [17], coagulation times for anticoagulation therapy [18] or assessment of symptoms of ulcerative colitis [15,16], and then forwarded to a server system which analyzes the data. This is performed by a decision support module located on the server. The system also provides data presentation and tailored feedback for the patient. A similar system design is described for the HeartCycle project by Reiter and Maglaveras [25] and by Biddiss et al [12]. The application domains are heart failure and coronary artery disease, and tailored advice is part of the approach Zheng et al. present the system structure of their *Personalised Self Management System* (PSMS) including a decision support ‘expert system’ [27,28]. The system is used e.g. for patients suffering from stroke, chronic pain and heart failure. The DS module supports two services: alerting and feedback, for example for exercises of the upper limb using inertial sensors [27].

Goldsmith et al. describe the Pediatric Cancer CareLink system and stress the importance of its decision support component for on-time assessment of symptoms and early detection of unfavorable or dangerous situations (e.g. medication side effects),but – as e.g. Abraham and Rosenthal [39] – do not focus on the DSS architecture in detail [40].

### Inclusion of context data and integration with existing HIS components

The above-mentioned HeartCycle project aims to record not only vital signs related to cardiovascular conditions, but also considers biochemical markers, questionnaires and other context information [25] for its decision support system. Bosworth et al. describe the design of an intervention trial which – amongst other interventions – employs a validated and already available DSS (ATHENA-HTN, http://www.openclinical.org/aisp_athena.html) to manage medication treatment in patients suffering from hypertension [14]. The DSS not only considers home-based blood pressure measurement data, but also additional clinical information from the Veteran’s Administrations electronic health record system, including laboratory results, diagnoses and previous blood pressure values.

Our research group has developed a DSS architecture that merges data from intelligent environments such as smart homes and vital signs data using the *Arden Syntax for Medical Logic Systems*, a standardized medical logic language [23,24]. The modular DSS has been implemented in a prototype for a scenario for exercise training of patients suffering from chronic obstructive pulmonary disease (COPD) using an OSGi middleware approach [21]. Within this context, we have developed a decision support module based on a dynamic Bayesian network that controls exercise training autonomously based on vital signs data [30], so that it may be used in an unsupervised training situation at home. A primary focus of our research is the integration of the DSS in existing or new health information system infrastructures, thus enabling to consider clinical context information such as diagnoses, laboratory findings or past test results as well as caregiver and patient information for the purpose of decision making [24].

The design as well as the implementation of an advanced DSS infrastructure are presented by Basilakis et al. in [9] and later in [10]. The system architecture integrates vital signs data with medications, patient questionnaire data and clinical data, and employs several different technologies for data analysis. ‘Input submodules’ perform data preprocessing and information extraction (e.g. using R scripts), followed by a rule-based interpretation using a professional production rules engine (JBoss) [9]. Established clinical guidelines serve as the basis for the production rules, which are formalized using XML technologies. The system can generate health status reports and alarms and has been tested for patients suffering from COPD and chronic heart failure [10].

### Requirements

Based on the above review of current literature and adding personal experience with the implementation of medical decision support systems, the author proposes the following requirements for such systems to be met:

· general:

real-time/ timely response and control [41]

alert prioritization [21,41]

 data safety and security as well as patient-controlled data transfer

· knowledge base (KB)/ medical logic:

 customization possible (‘prescription’ of modules [31]), no one-size-fits-all solution [24]

 adaptiveness, self-learning algorithms

 ‘intelligent’ data aggregation/ reduction, information extraction [11,42,43], prediction models [44-46]

 KB/ decision algorithms should be (easily) comprehensible; system should provide a user interface for editing, versioning and auditing

 standardized and modular representation of medical knowledge [24,26,37,47]

 should provide comprehensible explanations for the decisions taken

 possibility to translate/ incorporate clinical guidelines [10]

· integration:

 use of unified reference terminologies [9]

 implementation of standards for information representation from the health information system domain, for example HL7 Clinical Document Architecture (CDA) [21,43,47,48]

## Discussion

With the advent of health-enabling technologies, especially sensor technologies and advanced data analysis methods [11], their gradual integration as supportive technologies in current care scenarios on the one hand [49], and on the other hand by enabling new care services [27], a gradual permeation of these technologies into actual health care and patients’ homes beyond purely scientific limits can be observed. The properties identified above may serve to arrange current decision support systems into different categories with regard to autonomy, localization and integration in current health information system structures (aim#1). First studies have been published, proving the effectiveness of technology usage in specific disease management scenarios using home-recorded data, for example in patients with heart failure [50,51].

If the additional data that can be recorded using home-based or wearable technologies shall be used to generate more information resp. knowledge about a patient – and not lead to a data overload – the development of appropriate methods for analysis in terms of data reduction and information extraction are of paramount importance [52]. Too much unprocessed data may lead to confusion and even disregard of data, and not to shedding light into areas relevant for decision making in health matters. This counts not only for health care providers (physicians, nurses) but also for the patient herself or himself and her or his relatives [53]. Thus, ‘intelligent’ decision support systems or DS modules are necessary, and in fact are part of many system designs in the projects identified.

While the integration of solutions in health care settings is often ensured, details about the design of the decision support component, its contents and the methods used for decision making in most cases remain unclear. Furthermore, there is a lack of reports about the details of the DSS design infrastructure and about the formalization of the medical decision logic [30]. On a more general scale this also holds true for the architectures of so-called sensor-enhanced health information systems, of which decision supports systems are only one – albeit a crucial one – among many components [54]. Only few authors mention the integration of clinical knowledge sources such as institutional EHRs [10,21], which should be regarded as a prerequisite for ‘intelligent’ and individualized decision making [41], in analogy to the decision making process of a good general practitioner, who will always interpret data in the context of co-morbidities and co-medications, not to forget social context and personality. While the final decision about health-related measures should be taken by both the patient and the doctor as a consultant, a decision support module should be ‘intelligent’ enough to support this kind of decision process.

Many issues remain open considering decision support at home (DS@HOME). As mentioned before, there currently is no clear understanding about the way medical knowledge should be formalized for home care decision support. Most authors seem to use production rules. International standards for such logic, for example the Arden Syntax for Medical Logic Modules – for which an Open-Source compiler has recently been made available [55] – or GELLO [56], seem to be rarely – if at all – used. Without standardization, the exchange of decision components and their use beyond the specific scenario of a scientific project becomes very difficult. A similar development may be observed in clinical DSS which are often focused on specific problem areas and feature proprietary knowledge bases [8]. While the general system architecture of a DSS is obvious, many variations exist in terms of where the actual decision logic is located – in the personal resp. home environment [21,23] or in a centralized system (e.g. [12,17,18]). This has implications with regard to the updateability and customization of medical logic components in analogy to a physician’s prescription as proposed by Bott et al. [31], for example if the adaption of a medication dosage is necessary in a patient with chronic renal failure. Finally, the interfaces (if existent) to current clinical application systems containing the information (such as the diagnosis ICD-10 code *N18.2*, ‘the patient suffers from chronic renal failure’) which is necessary to interpret home-based data or to make decisions on this basis, currently seem neither widely accepted nor used.

The assembled requirements (aim#2) may serve developers of DSS as helpful guiding criteria for successful implementations, yet the author does not claim them to be complete or ranked according to their importance.

### Limitations

The author cannot rule out that the literature analysis is to some extent subjective. Many of the identified articles do not focus on the decision support components but rather on the overall system design and evaluation issues. Therefore, important facts about the DSS’s features and its implementation might not have been reported there and therefore may have been failed to be gathered in the review process. The results of the literature search as presented in Table 1 are ordered according to the first authors, yet some variations of a system have been described by different authors, for example by Finkelstein et al., Cross et al. and Joshi et al. While the basic system architecture remains the same, different application areas are addressed, and thus different decision components are used. Furthermore, the presentation does not make a difference between systems actually used in clinical practice and lab prototypes as e.g. in [24]. In addition to this, as the use of decision support systems in home environments has not found its way into large clinical trials so far, the author was not able to make a sound analysis of system architectures in terms of an outcome evaluation on the basis of this analysis.

## Conclusions

This paper focuses on decision support systems in home environments and presents the current state-of-the-art. Among the predominant challenges for current systems, integration with health information systems resp. clinical application systems have been stressed along with the need for the standardization of knowledge bases. The author has also identified a set of requirements for the successful implementation of DSS in home environments. In the future, these requirements will have to be met by system developers if the use of home-based health-enabling technologies shall be employed not only to gather large amounts of data, but also to provide a benefit for both the patient and the doctor by providing additional information that serves to enhance the knowledge basis on which decisions about health matters are finally made.

Further research is necessary with regard to the outcome of using decision support components at home respectively for home care. The research topics to be addressed should include cost-benefit-analyses, acceptance of DSS by patients as well as medical professionals, standardization of decision logic, pros and cons of different system architectures (centralized vs. locally distributed/ mobile) and methods of advanced individualized data analysis (data fusion, multi-parametric analyses).

## Competing interests

The author declares that he has no competing interests.

## Pre-publication history

The pre-publication history for this paper can be accessed here:

http://www.biomedcentral.com/1472-6947/12/43/prepub
